# AIV polyantigen epitope expressed by recombinant baculovirus induces a systemic immune response in chicken and mouse models

**DOI:** 10.1186/s12985-020-01388-w

**Published:** 2020-08-05

**Authors:** Lei Yu, Jun Pan, Guangli Cao, Mengsheng Jiang, Yunshan Zhang, Min Zhu, Zi Liang, Xing Zhang, Xiaolong Hu, Renyu Xue, Chengliang Gong

**Affiliations:** 1 School of Biology and Basic Medical Sciences, Soochow University, No.199 Ren’ai Road, Dushu Lake Higher Education Town, Suzhou Industrial Park, Suzhou, 215123 P.R. China; 2 Agricultural Biotechnology Research Institute, Agricultural biotechnology and Ecological Research Institute, Soochow University, Suzhou, 215123 China

**Keywords:** avian influenza virus, polyantigen epitope vaccine, immune response, baculovirus, chicken and mice

## Abstract

**Background:**

The protective efficacy of avian influenza virus (AIV) vaccines is unsatisfactory due to the presence of various serotypes generated by genetic reassortment. Thus, immunization with a polyantigen chimeric epitope vaccine may be an effective strategy for protecting poultry from infection with different AIV subtypes.

**Methods:**

Baculovirus has recently emerged as a novel and attractive gene delivery vehicle for animal cells. In the present study, a recombinant baculovirus BmNPV-CMV/THB-P10/CTLT containing a fused codon-optimized sequence (CTLT) of T lymphocyte epitopes from H1HA, H9HA, and H7HA AIV subtypes, and another fused codon-optimized sequence (THB) of Th and B cell epitopes from H1HA, H9HA, and H7HA AIV subtypes, driven by a baculovirus P10 promoter and cytomegalovirus CMV promoter, respectively, was constructed.

**Results:**

Western blotting and cellular immunofluorescence demonstrated that the CTLT (THB) can be expressed in rBac-CMV/THB-P10/CTLT-infected silkworm cells (mammalian HEK293T cells). Furthermore, the recombinant virus, rBac-CMV-THB-CTLT, was used to immunize both chickens and mice.

**Conclusions:**

The results of an indirect ELISA, immunohistochemistry, and T lymphocyte proliferation assay indicated that specific humoral and cellular responses were detected in both chicken and mice. These results suggest that rBac-CMV/THB-P10/CTLT can be developed as a potential vaccine against different AIV subtypes.

## Background

Avian influenza (AI) is caused by AI virus (AIV) infections and is one of the most important diseases affecting the poultry industry. There is increased concern due to the potential public health implications of AIV [31]. AIV contains eight single stranded, negative-sense RNA segments, and is a member of the genus influenza virus A of the *Orthomyxoviridae* family. Furthermore, there are 16 different hemagglutinins (H1–16) and 9 different neuraminidases (N1–9) among the AIV subtypes, which are characterized based on serological reactions [44, 46]. Moreover, AIVs are classified as either low (LP) or high pathogenicity (HP) according to differences in virulence [2]. Vaccination is one of the most efficient tools for preventing the emergence and transmission of AI. Since the protective response of the host to AIV are subtype-specific, a single AI vaccine cannot protect poultry from infections with various AIV subtypes [47].

To date, four technological approaches have been used to create AIV vaccines; inactivated whole AIV, the in vitro expression of AIV antigen proteins, in vivo expression of AIV antigen proteins and nucleic acids with an AIV antigen expression cassette [47] have been used to develop an AIV vaccine. An inactivated whole AIV vaccine has been widely used for over the past 30 years and accounts for 95.5% of AIV vaccine usage in poultry [45]; however, protective efficiency largely depends on whether the antigen of the AIV strain that was used for vaccine preparation matched the virus(es) circulating in the field [48]. Using reverse genetic systems for AIV [15, 33], custom-made inactivated AI vaccines that match circulating viruses can be created within a relative short period of time [48]. However, the use of inactivated AI vaccines is limited due to the high labor cost for intramuscular or subcutaneous vaccine injection.

Large quantities of AIV antigen protein can be expressed using mature prokaryotic, prokaryotic, or eukaryotic expression systems [13, 14, 42]. The purified recombinant protein can then be used as a vaccine following oil emulsification. The antigen can be produced in an in vitro system without safety concerns of growing AI vaccine viruses. Moreover, the chimeric gene that is joined in tandem by the epitopes from different antigens can be easily expressed using an in vitro system to produce a multivalent vaccine. Virus-like particles (VLPs) can be obtained by the self-assembly of viral structural proteins expressed in vitro, thereby inducing a sufficient immune response. Moreover, VLPs are non-infectious because they do not contain the viral genetic material. To date, several expression systems, including baculovirus [6], transformed cells [54], and plant systems [7], have been used to produce AIV VLPs. The VLPs produced with baculovirus, which comprise hemagglutinin, neuraminidase, and/or matrix protein M1 from a H5N1, have been shown to protect chickens from AIV infection [12, 37].

Vector-based vaccines are live-attenuated viruses modified by reverse genetics technology which contain the expression cassettes of the target antigen that can be expressed following the cellular entry of the vectors via infection, resulting in endogenous antigen processing and MHC class I restricted antigen presentation [10]. To date, adenovirus 5 [53], pseudorabies virus [26], fowl pox virus [49], Newcastle disease virus [23], herpesvirus of infectious laryngotracheitis virus [38], retrovirus [19], modified vaccinia ankara (MVA) virus [41], recombinant Newcastle disease viruses [25], and paramyxovirus [44, 46] are used as vector-based vaccines. Although live AIV vaccines are not available for use in animals, cold-adapted temperature-sensitive mutant AIV vaccines are safe for use in humans [43].

Nucleic acid-based vaccines are not associated with potentially hazardous pathogens, which can induce both a humoral and cellular immune response. Previous studies indicate that vaccination with the mRNA of hemagglutinin, neuraminidase, and nucleoproteins of the H1N1, H3N2, and H5N1 viruses can protect pigs, mice, and ferrets against clinical signs and reduce viral shedding in challenge experiments [39]. The ability to encode multiple genes of interest is a significant advantage of DNA-based vaccines; however, in contrast to mRNA-based vaccines, DNA-based vaccines are poorly transported into target cells [36]. Therefore, needle-mediated injections [30], intranasal administration [27], and electroporation [9], are used to deliver DNA vaccines into target cells. In addition, the safety of DNA vaccines is attracting attention, since DNA-based vaccines with selective markers (e.g., antibiotic resistance genes) can be integrated into the genomic DNA of host cells [52].

Baculoviruses are known to infect invertebrates, which has been widely applied for more than 30 years for the production of recombinant proteins in insect cells or larvae. Among the various baculoviruses, *Autographa californica* nucleopolyhedrovirus (AcMNPV) and *Bombyx mori* nucleopolyhedrovirus (BmNPV) are the most widely studied [21]. Moreover, a baculovirus expression vector system has been widely used for vaccine development due to the versatile features of baculoviruses, such as the large cloning capacity, post-translational modification in a eukaryotic system, replication-defect properties in mammalian cells, and broad tissue tropism [11]. It has also been reported that the HA1 protein of the H6 influenza virus and HA1 protein of the H5N1 AIV can be expressed in insect cells and *Spodoptera litura* larvae [18, 29]; indeed, the avian H7 influenza virus haemagglutinin was expressed in the silkworm (*B. mori*) pupa [32]. Additionally, recombinant subunit vaccines targeting haemagglutinin have been developed using a baculovirus expression vector system (BEVS). A trivalent recombinant HA influenza vaccine, Flublok®, was produced in insect cells using BEVS and subsequently approved in the United States [3]. In addition, baculovirus has been successfully applied for delivering foreign genes into mammalian cells without viral replication [11]. Baculovirus has also been reported to stimulate the host antiviral immune response in mammalian cells [4, 8] and to confer protection from lethal influenza virus infection in mice [1].

The protective efficacy of AIV vaccines is currently unsatisfactory due to the various serotypes that are generated by genetic reassortment; however, antigen epitopes can be modified and combined at the genetic level to develop a novel vaccine that can overcome the immune escape caused by viral mutations and improve the protective efficacy [50]. Therefore, vaccination with a polyantigen chimeric epitope vaccine may represent an effective strategy for protecting poultry from infection with various AIV subtypes. In the present study, a recombinant baculovirus rBac-CMV/THB-P10/CTLT containing a fused codon-optimized sequence (CTLT) of T cell epitopes from the hemagglutinin (HA) of H1, H9 and H7 AIV subtypes, and another fused codon-optimized sequence (THB) of Th and B cell epitope from HA of H1, H9, and H7 AIV subtypes, driven by a baculovirus P10 promoter and cytomegalovirus CMV promoter, respectively, was constructed. The results of the present study indicate that specific humoral and cellular responses can be detected in chickens and mice following the administration of rBac-CMV/THB-P10/CTLT, suggesting that rBac-CMV/THB-P10/CTLT can be developed as a potential vaccine against different AIV subtypes.

## Materials and methods

### Cell culture

BmN cells derived from silkworm (*B. mori*) ovaries were cultured in TC-100 medium supplemented with 10% fetal bovine serum (FBS) at 27°C. The human embryonic kidney 293 cell line (HEK293T), which was kindly provided by Professor Yuqing Zhang, School of Biology & Basic Medical Sciences, Soochow University, was cultured in complete DMEM culture medium at 37°C.

### Cytotoxic T lymphocyte (CTL) epitope prediction

Human leukocyte antigen (HLA)-A0201 and HLA-1101 were predicted by Bimas (http://bimas.dcrt.nih.gov/molbio/hla_bind/), SYFPEITHI (http://www.uni-tuebingen.de/uni/kxi/), Support Vector Machine (SVM) [5, 55], Artificial Neural Network (ANN) [5, 17] and Hidden Markov Model (HMM) [16], which presented cytotoxic T lymphocyte epitopes of hemagglutinin of A/NewCaledonia/20/99 (H1N1) (GenBank accession No. INA344014), A/Netherlands/127/03 (H7N7) (GenBank accession No. AAR02636) and A/swine/Shandong/nc/2005 (H9N2) (GenBank accession No. DQ997437) AIV subtypes. These subtypes are the highly pathogenic AIVs that were once epidemic in the world, therefore, these subtypes were selected as reference AIVs to explore whether vaccination with a polyantigen chimeric epitope vaccine can induce a systemic immune response in chicken and mouse models. Proteasomal cleavage prediction was performed with PAProC soft (http://www.paproc.de/) [34] to predict whether there are proteasomal cleavage sites in the predicted epitopes.

### Th and B cell (THB) epitope prediction

Th cell epitopes prediction was performed with SVM, ANN and HMM methods, B cell epitopes prediction was performed with BepiPred (http://www.cbs.dtu.dk/services/BepiPred/) [20] and PREDICTED ANTIGENIC PEPTIDES (http://imed.med.ucm.es/Tools/antigenic.pl) using A/NewCaledonia/20/99, A/Netherlands/127/03 and A/swine/Shandong/nc/2005 as reference strains.

### Design of expression cassette for multiple-epitope antigens

To construct the expression cassette of CTL multiple-epitope antigens (CTLT), matrix protein 1 and neuraminidase epitopes of AIV, a circumsporozoite VK210/VK247/Vivax-like epitopes fusion protein AdCh68-PvCSP (MGMQVQIQSLFLLLLWVPGSRG) (Sequence ID: AHC98633.1), the partial peptide sequence (KFVAAWTL) from ovarian cancer specific artificial polyepitopic immunogen of vaccinia virus (Sequence ID: AXN56537.1), the partial sequence (QYIKANSKFIGIT) from tetanus neurotoxin of *Clostridium tetani* (Sequence ID: WP_129031034.1), and the predicted CTL epitopes were used. The endoplasmic reticulum targeting signal and universal Th epitope (PAN-DR) were also integrated to the expression cassette; moreover, the each epitope was joined by an appropriate linker K/E/N/GAAA which was optimized according to proteasomal cleavage sites of the predicted epitope. In the expression cassette of CTLT, the amino acid sequences and conservation of selected CTL epitopes were shown in Table S1.

To construct the expression cassette of Th as well as B cell epitopes (THB), the matrix protein 1 epitopes of AIV, the partial sequence (EYLNKIQNSLSTEWSPCSVT) from chain A of circumsporozoite protein (Sequence ID: 3VDJ_A) of *Plasmodium falciparum* 3D7, circumsporozoite VK210/VK247/Vivax-like epitopes fusion protein AdCh68-PvCSP (MGMQVQIQSLFLLLLWVPGSRG) (Sequence ID: AHC98633.1), the peptide sequence (KRWIILGLNKIVRMY) from gag protein of human immunodeficiency virus 1 (Sequence ID:AHA33853.1), the partial peptide sequence (KFVAAWTL) from ovarian cancer specific artificial polyepitopic immunogen of vaccinia virus (Sequence ID: AXN56537.1), the peptide sequence (KAAAGGGGSGGGGSGGGGSID) from scFV antibody (Sequence ID: AFN94003.1) and the predicted Th as well B cell epitopes were used. Similarity, the endoplasmic reticulum targeting signal and universal Th epitope (PAN-DR) were also integrated to the expression cassette. Moreover, the each epitope from a AIV subtype was joined by a linker GPGPG to ensure that each epitope can function independently, the different subtype epitope sets were joined by linker KK to promote the correct cleavage of epitope boxes. In the expression cassette of THB, the amino acid sequences and conservation of selected THB epitopes were shown in Table S2.

### Synthesis of the sequences respectively coding CTLT and THB

CTLT (Figure S1) and THB (Figure S2) codon-optimized coding sequences based on the preference of the BmNPV codons were synthesized by GenScript Biotech Corp (Nanjiang, China), and cloned into the vector, pUC57-T (GenScript, Nanjiang, China), to generate pUC57-CTLT and pUC57-THB, respectively.

### Construction of recombinant plasmid pFastBac™DuaI-CMV-THB-CTLT

**Fig. 1:**
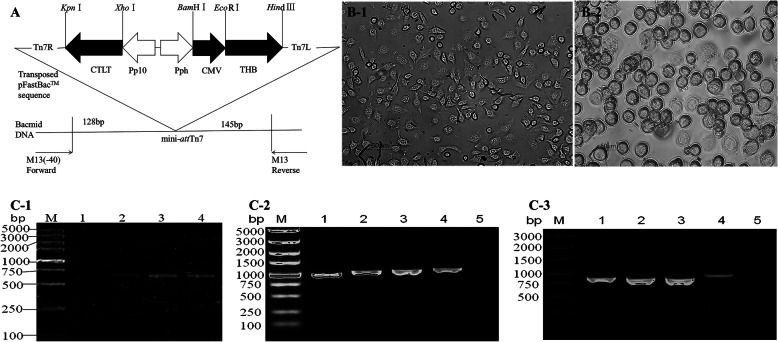
Construction of the Baculovirus transfer vector, pFastBac™DuaI-CMV/THB-P10/CTLT, and identification of the recombinant Baculovirus BmNPV-CMV/THB-P10/CTLT. A, Construction of the Baculovirus transfer vector, pFastBac™DuaI-CMV/THB-P10/CTLT, a coding sequence (CTLT) of T lymphocyte epitopes from H1HA, H9HA, and H7HA AIV subtypes was controlled by the baculovirus P10 promoter, and a coding sequence (THB) of B cell epitopes from the H1HA, H9HA, and H7HA AIV subtypes was driven by the CMV promoter. B-1, control BmN cells; B-2, the recombinant Baculovirus BmNPV-CMV/THB-P10/CTLT-infected BmN cells. C-1, the CMV promoter amplified from the recombinant Baculovirus BmNPV-CMV/THB-P10/CTLT; lane M: DNA marker; lane 1: BmNPV; and lanes 2–4: P1, P2, and P3 BmNPV-CMV/THB-P10/CTLT. C-2, the THB fragment amplified from the recombinant Baculovirus BmNPV-CMV/THB-P10/CTLT; lane M: DNA marker; lanes 1–3: P1, P2, and P3 BmNPV-CMV/THB-P10/CTLT; lane 4: pFastBacTMDuaI-CMV/THB-P10/CTLT; lane 5: BmNPV. C-3, the CTLT fragment amplified from the recombinant Baculovirus BmNPV-CMV/THB-P10/CTLT; lane M: DNA marker; lanes 1–3: P1, P2, and P3 BmNPV-CMV/THB-P10/CTLT; lane 4: pFastBacTMDuaI-CMV/THB-P10/CTLT; lane 5: BmNPV

The CMV promoter (0.6 kb) amplified from plasmid pcDNA3.1 (Invitrogen, Frederick, MD, USA) with the CMV-BI and CMV-EI primers (Table 1) was cloned into the *BamH*I /*Eco*RI sites of the pFastBac™Dual vector to generate pFastBac™Dual-CMV. The THB fragment (1.0 kb) excised from the plasmid pUC57-THB with *Eco*RI /*Hin*dIII was subcloned into pFastBac™Dual-CMV to generate a plasmid pFastBac™Dual-CMV-THB. Finally, the CTLT fragment (0.8 kb) excised from plasmid pUC57-CTLT with *Xho*I/*Kpn*I was inserted into the pFastBac™Dual-CMV/THB vector to generate the plasmid, pFastBac™DuaI-CMV/THB-P10/CTLT (Fig. 1).

**Table 1:** The primers used in this study

Primers	Sequences
CMV-BI	GGATCCGTTGACATTGATTATTGACTAG
CMV-EI	GAATTCGAGCTCTGCTTATATAGACCTCCC
M13 forward	CCCAGTCACGACGTTGTAAAACG
M13 reverse	AGCGGATAACAATTTCACACAGG
CTLT-XI2	CTCGAGATGGGTATGCAAGTTC
CTLT-KI2	GGTACCTTATCTCGGGATGTTTG
THB-EI2	GAATTCATGGGTATGCAGGTTC
THB-HD2	AAGCTTTTAGTCGATAGAGCCGCC
THB-F	AATTCATGGGATATGCAGGTTCAA
THB-R	GAGCAGGAAGCCGAAA
Actin-F	TCATGAAGTGTGACGTTGACATCCGT
Actin-R	CCTAGAAGCATTTGCGGTGCACGATG

### Generation of a recombinant baculovirus

The pFastBac™Dual-CMV/THB-P10/CTLT vector was transformed into *E. coli* DH10Bac/BmNPV provided by Prof. WB Wang of Jiangsu University to generate a recombinant Bacmid-CMV/THB-P10/CTLT using the Bac-To-Bac baculovirus expression system (Invitrogen, Frederick, MD, USA) following the manufacturer’s instructions. The recombinant Bacmid-CMV/THB-P10/CTLT (larger than 135 kb in size) was identified by PCR with the M13 forward and reverse primers (Table 1). These primers flanked the mini-attTn7 within the lacZ a-complementation region that harbor CTLT and THB (Fig. 1a).

The confirmed Bacmid-CMV/THB-P10/CTLT DNA was transfected into BmN cells using FuGENE HD Transfection Reagent (Roche, Indianapolis, Germany) to generate the recombinant baculovirus, BmNPV-CMV/THB-P10/CTLT. To further confirm the recombinant virus, DNA extracted from BmN cells infected with BmNPV-CMV/THB-P10/CTLT was used as a template. PCR was performed with CMV-BI/CMV-EI, CTLT-XI2/ CTLT-KI2, and THB-EI2/ THB-HD2 primers (Table 1) to amplify the CMV promoter, CTLT, and THB, respectively. Once the cells were confirmed to be infected, the virus from the cell culture was harvested as the P1 viral stock, and continuously proliferated through further infection in BmN cells until the P3 viral stock was obtained. The stock was stored at 4°C in the dark.

### Inoculation of virus

A total of 2 × 10^5^ HEK293T or BmN cells (1 mL) were inoculated with 100 μL of BmNPV-CMV/THB-P10/CTLT (TCID_50_ = 10^− 11^/100 μL) after washing twice in 1 × PBS, and were subsequently cultured in complete DMEM or TC-100 culture medium, respectively.

### Detection of BmNPV-CMV/THB-P10/CTLT by PCR

The BmNPV-CMV/THB-P10/CTLT was inoculated into HEK293T or BmN cells, which were collected at 72 h post-inoculation for the extraction of genomic DNA and total RNA. The genomic DNA was used as a template for the amplification of the CMV promoter, THB, and CTLT fragments with primer pairs CMV-BI/CMV-EI, THB-EI2/THB-HD2, and CTLT-XI2/CTLT-KI2, respectively. THB and CTLT transcription was detected by PCR with the primer pairs, THB-EI2/THB-HD2 and CTLT-XI2/CTLT-KI2, after the extracted RNA was transcribed into cDNA.

### qRT-PCR

HEK293T cells (2 × 10^5^) were infected with BmNPV-CMV/THB-P10/CTLT (10 μL; 10^11^ TCID_50_), and collected at 0, 12, 24, 48, and 72 h post-infection. The total RNA was extracted. After being reverse transcribed into cDNA, qPCR was performed with the primer pairs, THB-F and THB-R (Table 1), to determine the level of THB expression. The beta-actin gene was used as an internal reference gene. The experiment was repeated three times.

### Western blot

To detect the level of CTLT and THB protein expression, BmN and HEK293T cells were collected at 120 h post-inoculation, and subjected to SDS-PAGE. The proteins on the PAGE gel were transferred onto a polyvinylidene difluoride membrane, and a Western blot was carried out with the primary antibody of a mouse anti-H7H9 HA (1:2000) (Sino Biologocal Inc., Peking, China) and the second antibody of a HRP-conjugated goat anti-mouse IgG (1:20000) (Sino Biologocal Inc., Peking, China). The uninoculated cells were used as a negative control.

### Immunofluorescence assay

BmN and HEK293T cells were collected at 120 h post-inoculation. The cells were fixed with 4% paraformaldehyde, and an immunofluorescence assay was performed following labeling with mouse anti-H7H9 HA (1:100) and a FITC-conjugated goat anti-mouse IgG (1:500) (Bio-world, Dublin, USA). The cells incubated with pre-immune antiserum were used as a negative control. After the uncombined goat anti-mouse IgG antibody was removed, the cells were stained with 4′,6-diamidino-2-phenylindole (DAPI), and examined under a fluorescent microscope (Leica Microsystems, Mannheim, Germany).

### Animal immunization

The chickens were ordered from Wujiang Jinjiaba Red Star Chicken Farm, Suzhou city, China. Chickens aged 5 weeks old were immunized by intraperitoneal injection with 10^11^ TCID_50_ (100 μL), 5 × 10^10^ TCID_50_ (100 μL), and 10^10^ TCID_50_ (100 μL) of BmNPV-CMV/THB-P10/CTLT in a sodium butyrate solution (4 mol/L).

The mice were provided by Experimental Animal Center of Soochow University. Specific pathogen-free (SPF) male BALB/c mice (approximately 20 g) were immunized by intravenous tail vein injection with 10^10^ TCID_50_, 5 × 10^9^ TCID_50_, 10^9^ TCID_50_ of BmNPV-CMV/THB-P10/CTLT in a sodium butyrate solution (4 mol/L). The experimental work was approved by the Committee on the Ethics of Animal Experiments of Soochow University, and the reference number of the Ethics Committee was 201,605,328.

### Blood sampling, serum preparation and determination of the antibody titer

The blood collected from the chickens and mice was placed in an EP tube with 10 μL of heparin sodium (10%). After 2 h post-sampling, the blood was centrifuged at 6000×g for 8 min. The supernatant was collected and stored as the serum. The antibody titer in the serum of the immunized animals was determined using an ELISA.

### Splenic T lymphocyte proliferative responses in chickens

Five-week-old chickens were immunized twice by intraperitoneal injection with BmNPV-CMV/THB-P10/CTLT 7 days apart. After 14 days, the spleens were dissected from the immunized chickens and processed to create single-cell suspensions. A splenic T lymphocyte proliferative response assay was performed as described previously [51]. Briefly, the lymphocyte cell suspension (1 × 10^7^ cells/mL) was stimulated with ConA (5 μg/mL final concentration) and H7 standard antigen (0.5 μg/mL final concentration) (Sino Biologocal Inc., Peking, China) was seeded into a 96-well plate at 100 μL per well. An unstimulated cellular suspension was used as a control. The cells were incubated at 5% CO_2_ and 39.5 °C for 44 h, and the plate was incubated with 20 μL MTT solution (5 mg/mL) for 4 h. After removing the culture medium from each well, 100 μL of the lysate (10% SDS in 0.01 M HCl) was added and the cells were incubated for a further 2 h before a spectrophotometric measurement was taken at 570 nm. The lymphocyte transformation was judged by the stimulation index (SI) (SI = T_A570_ / C_A570_, in which T_A570_ was an absorbance measured at 570 nm for the test wells, and C_A570_ was an absorbance measured at 570 nm for the control well). The test was repeated four times.

### Organ coefficient

SPF male BALB/c mice (approximately 20 g) were intraperitoneally injected with BmNPV-CMV/THB-P10/CTLT (100 μL) at a dose of 10^12^ TCID_50_. The organ coefficient of the spleens was investigated at 48 h post-injection. Unimmunized mice were used as a control. The experiment was repeated three times.

## Results

### Epitope prediction and design of multiple-epitope antigens expression cassette

HLA-A0201 and HLA-1101 presented CTL epitopes of H1N1, H7N7 and H9N2 AIV subtypes were predicted by Bimas, SYFPEITHI, SVM, ANN, and HMM to design the multiple-epitope antigens expression cassette of CTLT (Figure S1), the results showed that H1HA 42-50aa (CLLKGIAPLN), H1HA 100-108aa (ELREQLSSV), H1HA132–140 (VTAACSHAG), H9HA 19-27aa (TLTENNVPV), H9HA 79-87aa (YIVERPSAV), H9HA 124–132 aa (NVSYSGTSK), H7HA 26-34aa (TLTERGVEV), H7HA 192-200aa (KLYGSGSKL) and H7HA 40–48 aa (TVERTNIPR) common CTL epitopes predicted by different methods could be used. The conservation of selected CTL epitopes in different AIV subtypes was indicated in Table S1. Th cell epitopes prediction was performed with SVM, ANN and HMM methods, B cell epitopes prediction was performed with BepiPred and PREDICTED ANTIGENIC PEPTIDES soft, the results showed that H1HA188–205 aa (RALYHTENAYVSVVSSHY), H1HA 78-87aa (KESWSYIVETPNPEN), H1NA 125-139aa (NHTVTGVSASCSHNG), H9HA 123–140 aa (NVSYSGTSKACSDSFYRS), H9HA 74-91aa (GGKWSYIVERPSAVNGMC), H9HA 38-55aa (HNGMLCATNLGHPLILNT), H7HA 173-189aa (DPALIIWGIHHSGSTAE), H7HA 263-277aa(SMGIQSDVQVDANCE), and H7HA 190-204aa (QTKLYGSGSKLITVG) were Th cell epitopes as well as B cell epitopes, therefore these epitopes were used to design the expression cassette of THB (Figure S2). The conservation of selected THB epitopes in different AIV subtypes was indicated in Table S1. To ensure that each epitope can function independently, the each epitope was joined by a linker K/E/N/GAAA which was optimized according to proteasomal cleavage sites of the predicted epitope in the CTLT expression cassette. Epitopes from same AIV subtype was joined by linker GPGPG, and different subtype epitope sets was joined by linker KK in the THB expression cassette, respectively. Moreover, the collected the matrix protein 1 and neuraminidase epitopes of AIV, the helper epitopes to major histocompatibility complex (MHC)-restricted common epitopes and endoplasmic reticulum signal sequence were also used to design the CTLT and THB expression cassettes.

### Identification of the recombinant bacmid and recombinant virus

To obtain an AIV polyantigen epitope vaccine based on a baculovirus vector, the synthesized CTLT (Figure S1) and THB (Figure S2) coding sequences which were optimized according to the preference of the BmNPV codons were cloned into a vector to generate pUC57-CTLT and pUC57-THB, respectively. Next, the baculovirus transfer vector pFastBac™DuaI-CMV-THB-CTLT, in which CTLT and THB, driven by a baculovirus P10 promoter and cytomegalovirus CMV promoter, respectively, was constructed. To generate the recombinant bacmid, the pFastBac™DuaI-CMV-THB-CTLT was transformed into *E. coli* DH10Bac. To identify the recombinant bacmid, DNA extracted from white colonies was used as a template, and PCR was performed with M13 forward and M13 reverse primers. A 4.96 kb product that was consistent with the theoretical molecular weight, could be detected (Data not shown), suggesting that the THB and CTLT expression cassettes had integrated into the bacmid genomic DNA. The generated recombinant was termed Bacmid-CMV/THB-P10/CTLT. To generate a recombinant baculovirus, the bacmid-CMV/THB-P10/CTLT was transfected into cultured BmN cells. The transfected cells typically exhibited an enlarged cell volume and size of the nuclei, ceased growing, became rounded, and exhibited detachment and lysis at 72 h post-transfection (Fig. 1b). The DNA extracted from the transfected cells was identified by PCR with the primer pairs, CMV-BI/CMV-EI, THB-EI2/THB-HD2, and CTLT-XI2/CTLT-KI2, respectively. The PCR products representing the CMV promoter (0.5 kb), THB fragment (1.0 kb), and CTLT fragment (0.8 kb) could be detected, indicating that the recombinant BmNPV-CMV/THB-P10/CTLT was successfully constructed (Fig. 1c). After 3 cycles of infection, a high titer P3 viral stock was collected and preserved in the dark at 4°C.

### Expression of antigen epitopes in BmN and HEK293T cells infected with recombinant baculovirus, BmNPV-CMV/THB-P10/CTLT

**Fig. 2:**
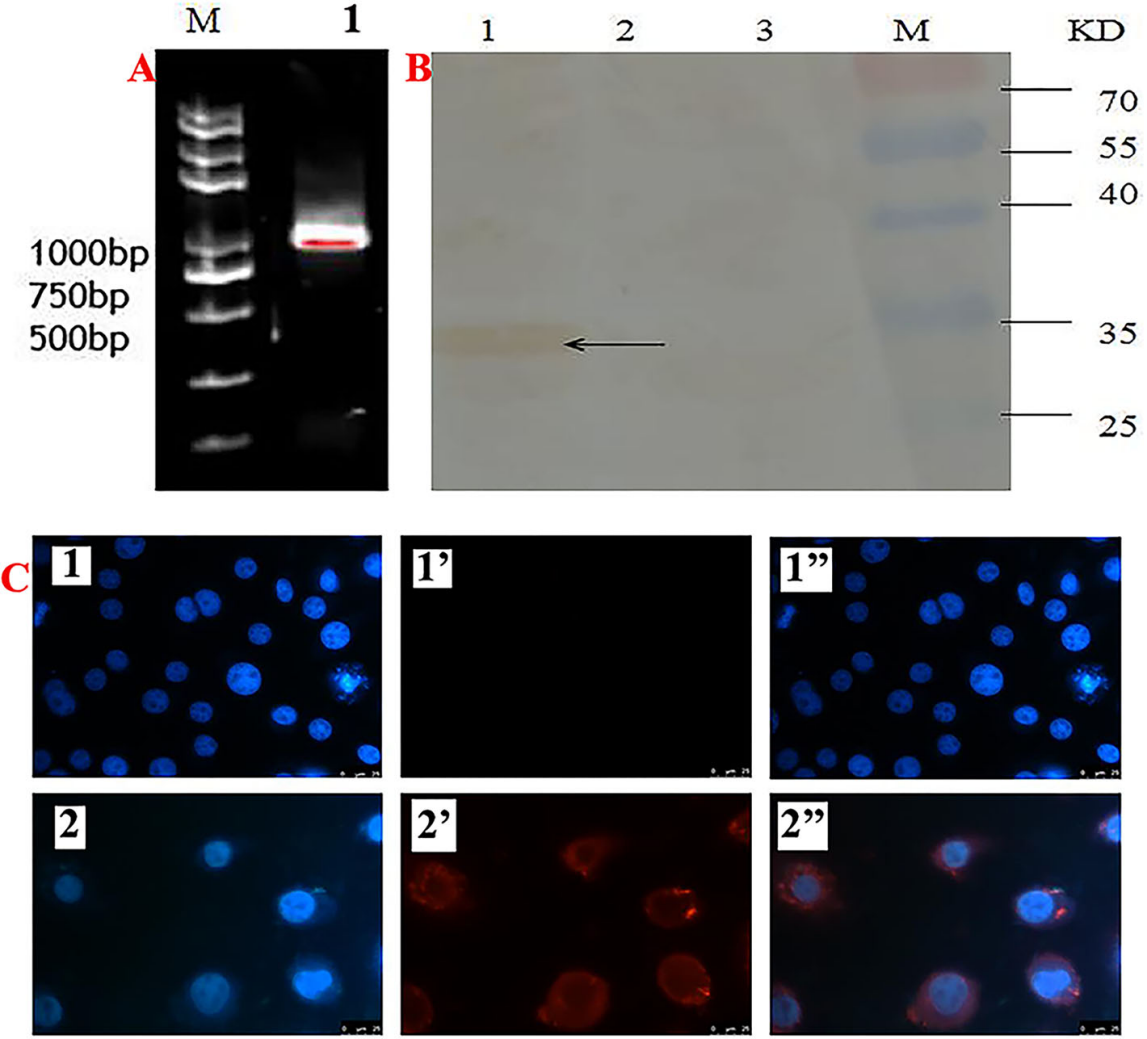
CTLT expressed in BmNPV-CMV/THB-P10/CTLT-infected BmN cells. **a** CTLT transcript detected by RT-PCR; Lane M: DNA marker; Lane 1: CTLT transcript. **b** CTLT expressed in the BmNPV-CMV/THB-P10/CTLT-infected BmN cells was detected by Western blot; lane 1: BmN cells infected with BmNPV-CMV/THB-P10/CTLT; lane 2: BmN cells infected with BmNPV; lane 3: normal BmN cells. Primary antibody; lane M: protein marker. Mouse anti-H7H9 HA was used as a primary antibody; HRP-conjugated goat anti-mouse IgG was used as a secondary antibody. **c**, CTLT expressed in BmNPV-CMV/THB-P10/CTLT-infected BmN cells was detected by immunohistochemistry. 1, 1′ and 1″, normal BmN cells; 2, 2’and 2″, BmNPV-CMV/THB-P10/CTLT infected BmN cells; 1 and 2, the cell nucleus was stained by DAPI; 1′ and 2′, CTLT stained by Cy3; 1″, the merger of 1 and 1′; 2″, the merger of 2 and 2′. Mouse anti-H7H9 HA was used as a primary antibody; Cy3-labeled goat anti-mouse IgG was used as a secondary antibody

To confirm the expression of epitopes in the BmNPV-CMV/THB-P10/CTLT-infected BmN cells, RT-PCR was performed to detect CTLT transcription controlled by the p10 promoter. The results showed that a specific PCR product representing the transcript of CTLT was observed (Fig. 2a). Moreover, the CTLT expressed in the BmNPV-CMV/THB-P10/CTLT-infected BmN cells could be also detected by Western blot (Fig. 2b) and immunocytochemistry (Fig. 2c), indicating that CTLT controlled by the p10 promoter was expressed in the BmN cells.

**Fig. 3:**
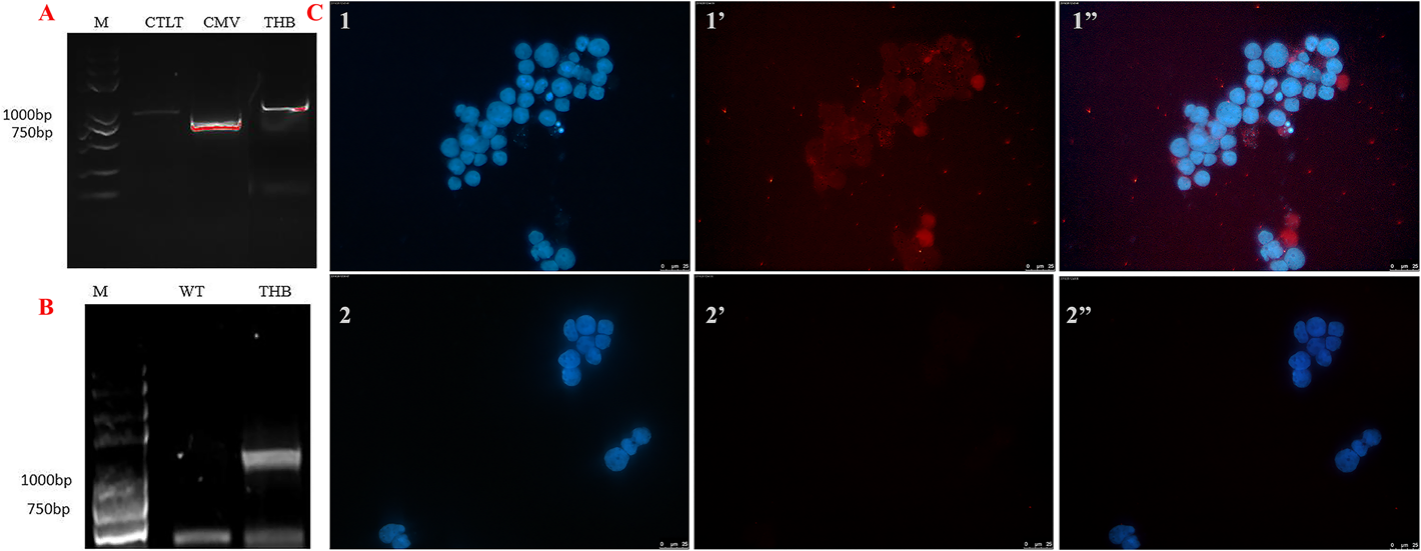
THB expression in BmNPV-CMV/THB-P10/CTLT-infected HEK293T cells. **a** PCR detection of the BmNPV-CMV/THB-P10/CTLT in the HECK293T cells. Lane M: DNA marker; Lane CTLT: PCR product of CTLC; Lane CMV: PCR product of the CMV promoter; lane THB: PCR product of THB. **b** THB transcript detected by RT-PCR. Lane WT: BmNPV-infected HECK293T cells; lane THB: BmNPV-CMV/THB-P10/CTLT-infected HECK293T cells. **c** The expression of THB in the BmNPV-CMV/THB-P10/CTLT-infected HEK293T cells was detected by immunohistochemistry. 1, 1′ and 1″, BmNPV-CMV/THB-P10/CTLT-infected BmN cells; 2, 2’and 2″, normal BmN cells; 1 and 2, cell nucleus was stained by DAPI; 1′ and 2′, the THB stained by Cy3; 1″, the merger of 1 and 1′; 2″, the merger of 2 and 2′. Mouse anti-H7H9 HA was used as the primary antibody; Cy3-labbled goat anti-mouse IgG was used as a secondary antibody

To confirm that the HEK293T cells could be transduced by BmNPV-CMV/THB-P10/CTLT, DNA extracted from the HEK293T cells inoculated with BmNPV-CMV/THB-P10/CTLT was used as a template to amplify the CMV promoter, THB fragment, and CTLT fragment by PCR with primer pairs CMV-BI/CMV-EI, THB-EI2/THB-HD2, and CTLT-XI2/CTLT-KI2, respectively. The PCR products representing the CMV promoter, THB fragment, and CTLT fragment could be detected (Fig. 3a), indicating that BmNPV-CMV/THB-P10/CTLT could enter into the HEK293T cells. Moreover, to detect the transcription of THB controlled by the CMV promoter, PCR was performed after the total RNA extracted from BmNPV-CMV/THB-P10/CTLT-infected HEK293T cells was reverse transcribed into cDNA; a specific product representing the THB transcript was detected (Fig. 3b), indicating that the THB antigen epitopes were transcribed. Furthermore, immunocytochemistry was used to detect the expression of THB, with red fluorescence representing THB observed in the BmNPV-CMV/THB-P10/CTLT-infected HEK293T cells, suggesting that THB was expressed (Fig. 3c). qRT-PCR was performed to determine the level of THB expression in the infected cells at different stages. The results showed that the level of THB expression increased with infection, peaking at 48 h post-infection (Figure S3).

### A specific immune response against THB was generated in chickens vaccinated with BmNPV-CMV/THB-P10/CTLT

**Fig. 4:**
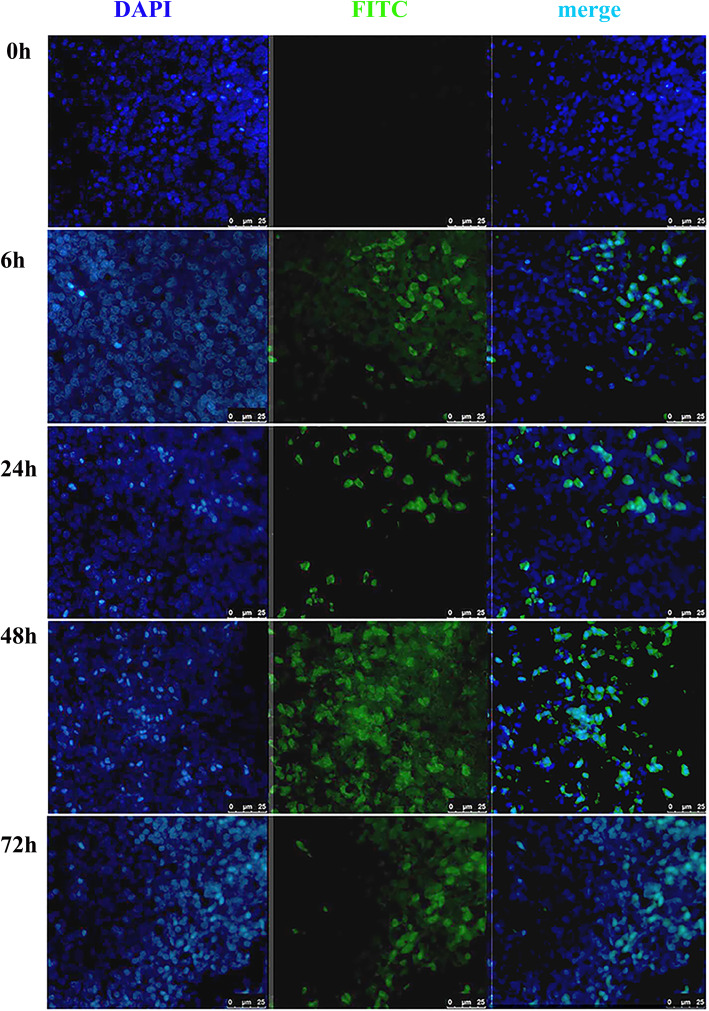
THB expressed in the splenocytes of BmNPV-CMV/THB-P10/CTLT-injected chickens. The spleens were dissected from the chickens at 0, 6, 24, 48, and 72 h post-injection with BmNPV-CMV/THB-P10/CTLT, and immunohistochemistry was carried out with the mouse anti-H7H9 HA (primary antibody) and FITC-labeled goat anti-mouse IgG (secondary antibody). The cell nucleus was stained with DAPI

To determine whether BmNPV-CMV/THB-P10/CTLT could deliver the foreign gene into the chickens, the spleens were dissected from the chickens at 0, 6, 24, 48, and 72 h post-injection with BmNPV-CMV/THB-P10/CTLT, and immunohistochemistry was performed to detect the expression of THB in the spleen. The results showed that green fluorescence, representing THB expression, could be observed in the spleens collected at 6 h post-injection. The fluorescence intensity peaked at 48 h post-injection, and subsequently decreased at 72 h post-injection (Fig. 4), suggesting that the THB expression cassette was delivered to the splenocytes and expressed.

**Fig. 5:**
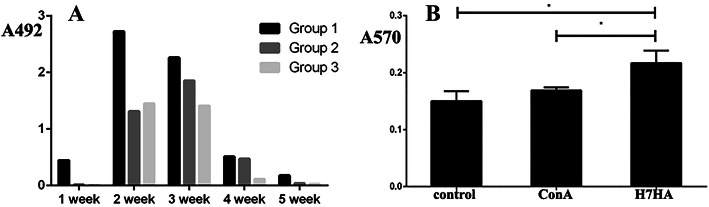
A specific immune response against THB could be generated in the chickens vaccinated with BmNPV-CMV/THB-P10/CTLT. **a** The detected levels of anti-THB antibodies in the serum of the vaccinated chickens with BmNPV-CMV-THB-CTLT. The chickens were vaccinated with BmNPV-CMV-THB-CTLT at a dose of 10^12^ TCID_50_/kg (Group 1), 5 × 10^11^ TCID_50_ /kg (Group 2), and 10^11^ TCID_50_/kg (Group 3). The serum samples at 1, 2, 3, 4, and 5 weeks post-vaccination were used to determine the level of anti-THB antibodies via an indirect ELISA. **b** The splenic T lymphocyte proliferative responses in vaccinated chickens. The lymphocyte cell suspension was stimulated with ConA (5 μg/mL final concentration) and H7HA standard antigen (0.5 μg/mL final concentration) was seeded into a 96-well plate at 100 μL per well. An unstimulated cellular suspension was used as a control. The cells were incubated at 5% CO_2_ and 39.5 °C for 44 h. The cell number was determined using the MTT method. *P < 0.05

To determine whether BmNPV-CMV-THB-CTLT can induce a specific humoral immune response against THB antigen expression in the chickens, an indirect ELISA was used to determine the level of H7HA antibody in the chicken serum. The results showed that antibodies could be detected at 1 week post-vaccination in the chickens injected with BmNPV-CMV-THB-CTLT at a dose of 10^12^ TCID_50_/kg; the antibody levels increased at 2 weeks post-vaccination, and subsequently decreased. In the chickens injected with a dose of 10^11^ TCID_50_/kg and 10^10^ TCID_50_/kg, the antibody levels peaked at 3 weeks post-vaccination, and subsequently decreased. Moreover, the antibody response increased following immunization in a dose-dependent manner (Fig. 5a).

The results of the splenic T lymphocyte proliferative response in the chickens showed that the absorbance value (A_570_) at 570 nm for the H7HA and ConA irritants was larger compared to the negative control, suggesting a specific cellular immune response against H7HA was induced when the chickens were vaccinated with BmNPV-CMV-THB-CTLT (Fig. 5b).

### Specific immune response against THB could be generated in the mice vaccinated with BmNPV-CMV/THB-P10/CTLT

**Fig. 6:**
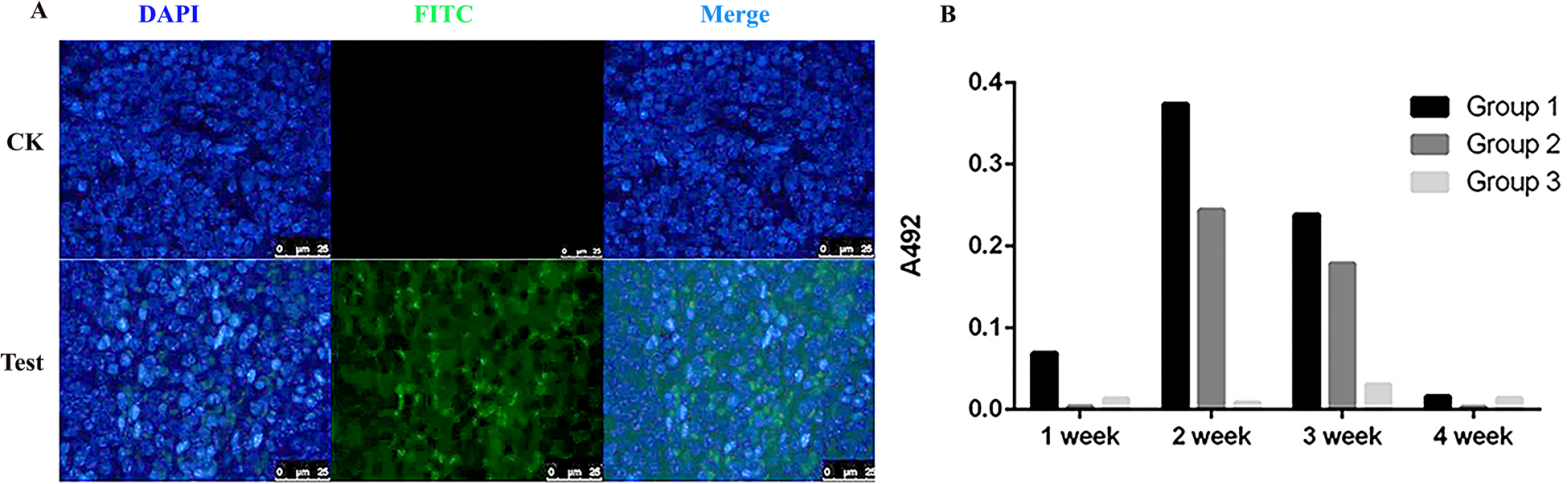
THB expressed in the splenocytes of the BmNPV-CMV/THB-P10/CTLT-injected mice and the level of anti-THB antibody detection. **a** THB expressed in mouse splenocytes. The spleens were dissected from the mice at 48 h post-vaccination with BmNPV-CMV/THB-P10/CTLT, and immunohistochemistry was performed with the mouse anti-H7H9 HA (primary antibody) and FITC-labeled goat anti-mouse IgG (secondary antibody). The cell nucleus was stained with DAPI. CK, normal mouse; Test, the mice vaccinated with BmNPV-CMV/THB-P10/CTLT. **b** The level of anti-THB antibody detected in the serum of the vaccinated mice with BmNPV-CMV-THB-CTLT. The mice were vaccinated with BmNPV-CMV-THB-CTLT at a dose of 10^10^ TCID_50_/kg (Group 1), 5 × 10^9^ TCID_50_ /kg (Group 2), and 10^9^ TCID_50_/kg (Group 3). The serum sampled at 1, 2, 3, and 4 weeks post-vaccination were used to determine the level of anti-THB antibodies by indirect ELISA

To explore whether the foreign gene could be delivered into mice by the recombinant baculovirus BmNPV-CMV/THB-P10-CTLT, immunofluorescence of the spleens harvested from mice at 48 h post-tail vein injection with BmNPV-CMV/THB-P10-CTLT was performed with an anti-H7HN antibody. The results showed that green fluorescence could be detected, suggesting that the THB expression cassette was delivered into the spleen and expressed (Fig. 6a).

Moreover, indirect ELISA results showed that anti-H7HA-specific antibodies could be detected at 1 week post-vaccination with BmNPV-CMV-THB-CTLT at a dose of 10^10^ TCID_50_ (Group 1); the antibody levels peaked at 2 weeks post-vaccination, and subsequently decreased. Similar changes in the antibody levels could be found in the mice vaccinated with BmNPV-CMV-THB-CTLT at a dose of 5 × 10^9^ TCID_50_ (Group 2). However, in the mice vaccinated with a dose of 10^9^ TCID_50_ (Group 3), the antibody levels were lower (Fig. 6b).

### The organ coefficient of the spleens increased after the mice were vaccinated with BmNPV-CMV-THB-CTLT

The mice were immunized with BmNPV-CMV/THB-P10/CTLT, and the organ coefficient of the spleens was investigated at 48 h post-vaccination. The results showed that the viscera coefficients of the immunized mice were increased compared to unimmunized mice (Figure S4).

## Discussion

To date, the widely used AIV vaccine is an inactivated whole AIV vaccine; however, its protective efficacy is unsatisfactory [45]. Therefore, substantial effort has been placed on the development of novel types of vaccines using various technological approaches. Among these approaches, using expressed AIV antigens, chimeric epitopes, or an in vitro self-assembly VLP system are the most popular vaccine platforms. Furthermore, adenovirus [53], pseudorabies virus [26], fowl pox virus [49], Newcastle Disease virus [23, 25], herpesviruses [38], retroviruses [19], MVA virus (Rahn et al, [41]), and paramyxovirus [44, 46] have been used as vector-based vaccines to deliver AIV antigen genes into target cells.

Baculovirus is a type of invertebrate virus. The classical swine fever vaccine, Porcilis Pesti™ (Merck, www.merck.com) expressed with BEVS was commercially approved in 2010. In addition, baculoviruses, including AcMNPV and BmNPV, have been used to express AVI antigens ([12, 18, 29, 32, 37]; Balraj [3]). The influenza vaccines, Provenge™ (Dendreon, www.dendreon.com) and FluBlok™ (Protein Sciences Corporation, www.proteinsciences.com), produced by baculovirus were approved by the Food and Drug Administration in 2010 and 2013. Currently, baculoviruses with replication-defect properties and broad tissue tropism have been used to deliver genes into animal cells [1, 4]. Moreover, it was reported that the BmNPV vector is more stable against complement inactivation in human serum than the AcMNPV vector [28]. Therefore, in the present study, BmNPV was used to deliver AIV polyantigen chimeric epitopes genes into chickens and mice to assess the efficacy of a baculovirus-vectored AIV vaccine.

The protective efficacy of an AIV vaccine largely depends on whether the antigen of the AIV strain used for vaccine preparation matches the virus(es) circulating in the field [48]. Thus, a single AI vaccine cannot protect poultry from infection with all of the AIV subtypes [47]. Moreover, various AIV serotypes are generated by genetic reassortment. Therefore, the recombinant baculovirus, BmNPV-CMV/THB-P10/CTLT, simultaneously containing CTLT of the T lymphocyte epitopes from the H1HA, H9HA, and H7HA AIV subtypes, and another THB of the Th and B cell epitopes from H1HA, H9HA, and H7HA AIV subtypes was constructed in this study. PCR, RT-PCR, and immunocytochemistry confirmed that BmNPV-CMV/THB-P10/CTLT could enter into the HEK293T cells, and the THB gene driven by the CMV promoter was expressed, indicating that the THB gene was delivered into the HEK293T cells. In addition, qRT-PCR showed that the level of THB expression peaked in HEK293T cells at 48 h post-transduction with BmNPV-CMV/THB-P10/CTLT, and subsequently decreased, since the baculovirus could not replicate in the invertebrate animal cells (Balraj [3]).

Previous studies indicate that the BmNPV vector can deliver reporter genes into different tissues and organs in mice and chicks [28]. Similar results were observed in the present study, indicating that BmNPV could be used as a gene delivery vehicle for animals. Moreover, specific humoral and cellular immune responses against the expressed THB antigen were detected in both chickens and mice, suggesting that BmNPV-CMV/THB-P10/CTLT has the potential for the development of a vector-based vaccine against AIV. The ELISA results indicate that the antibody levels induced by BmNPV-CMV/THB-P10/CTLT increased roughly with the immunization in a dose-dependent manner in both chickens and mice. Although previous studies have shown that the epitope regions selected in this study are critical for the production of neutralizing antibodies [22, 24, 35, 40], a neutralizing assay for AIV should be conducted to test whether these B cell epitopes could induce neutralized antibody in the further.

The use of inactivated AI vaccines is limited due to the high labor cost for intramuscular or subcutaneous injection of the vaccines. Previous studies indicate that the gene could be delivered into tissues and organs in mice and chickens via the intragastric administration of the BmNPV vector [28]. Moreover, it was reported that H7 VLPs could produce hemagglutination inhibition antibody in chickens and mice following oral immunization [32]. In the present study, CTLT driven by the P10 promoter could be expressed in BmNPV-CMV/THB-P10/CTLT-infected silkworm BmN cells, but whether the specific immune response can be induced in mice and chickens via the intragastric administration of the BmNPV-CMV/THB-P10/CTLT or oral immunization with the BmNPV-CMV/THB-P10/CTLT-infected silkworms lyophilized powder containing BmNPV-CMV/THB-P10/CTLT and recombinant CTLT, need further research. Moreover, although specific immune response could be induced in the mice and chickens vaccinated with BmNPV-CMV/THB-P10/CTLT, whether protective immunity against AIVs can be provided, further investigation is also needed.

## Conclusions

The results of an indirect ELISA, immunohistochemistry, and T lymphocyte proliferation assay indicated that specific humoral and cellular responses were detected in both chicken and mice. These results suggest that rBac-CMV/THB-P10/CTLT can be developed as a potential vaccine against different AIV subtypes.

## Supplementary information

**Additional file 1: Figure S1. The synthesized CTLT sequence.**

**Additional file 2: Figure S2. The synthesized THB sequence.**

**Additional file 3: Figure S3.** Level of THB expression at different stages in BmNPV-CMV/THB-P10/CTLT-infected HEK293T cells. The level of THB expression at 0, 12, 24, 48, and 72 h post-infection was determined by qRT-PCR.

**Additional file 4: Figure S4.** Effect of vaccination with BmNPV-CMV-THB-CTLT on the organ coefficient of the spleen. SPF BALB/c mice (approximately 20 g) were intraperitoneally injected with BmNPV-CMV/THB-P10/CTLT (100 μL) at a dose of 10^12^ TCID_50_, The organ coefficient of the spleens was investigated at 48 h post-injection. The unimmunized mice were used as a control. *p < 0.05.

**Additional file 5: Table S1.** The amino acid sequences and conservation of selected CTL epitopes.

**Additional file 6: Table S2.** The amino acid sequences and conservation of selected THB epitopes.

## Data Availability

The synthesized sequence of CTLT according to the codon preference of the BmNPV (GenBank accession numbers: MN533977). Predicted coding sequence of THB according to the codon preference of the BmNPV (GenBank accession numbers: MN533978).

## Abbreviations

AI: Avian influenza
AIV: Avian influenza virus
VLPs: Virus-like particles
AcMNPV: *Autographa californica* nucleopolyhedrovirus
BmNPV: *Bombyx mori* nucleopolyhedrovirus
BEVS: Baculovirus expression vector system
CTL: Cytotoxic T lymphocyte
THB: Th and B cell
