# Supplementary figures and images for: AIV polyantigen epitope expressed by recombinant baculovirus induces a systemic immune response in chicken and mouse models
Source: Virol J. 2020 Aug 5;17:121. doi: 10.1186/s12985-020-01388-w (PMC7403573; doi:10.1186/s12985-020-01388-w)

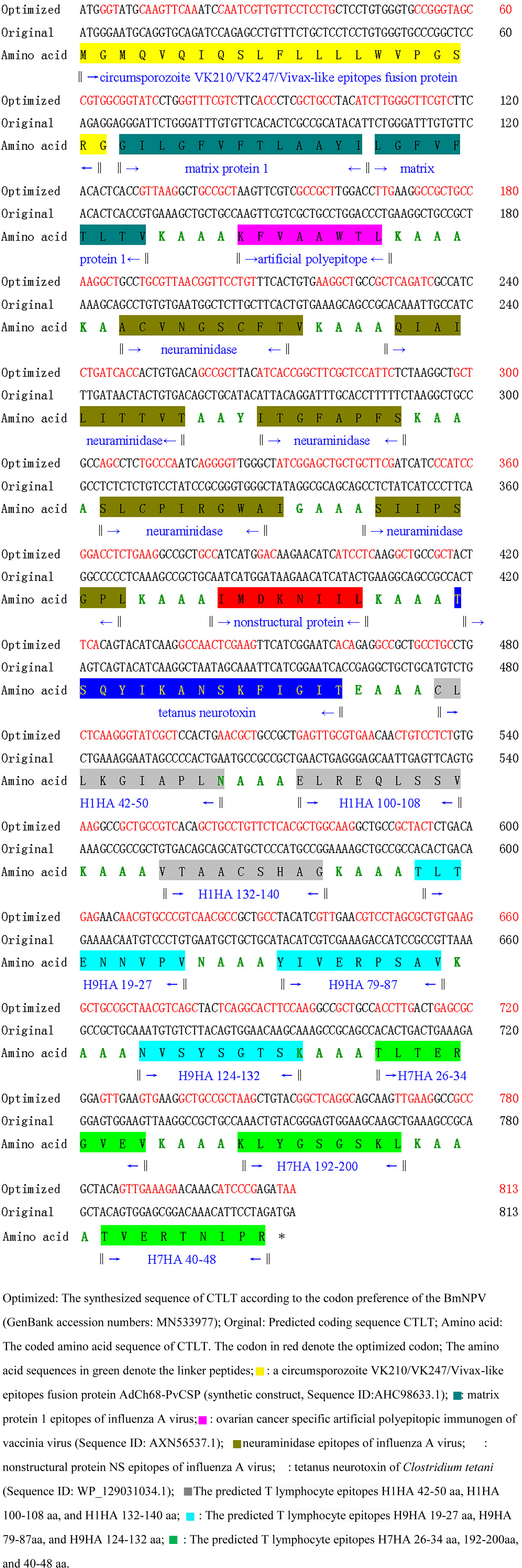

Supplement: Supplementary file 1 — Additional file 1: Figure S1. The synthesized CTLT sequence. [file 12985_2020_1388_MOESM1_ESM.tif]

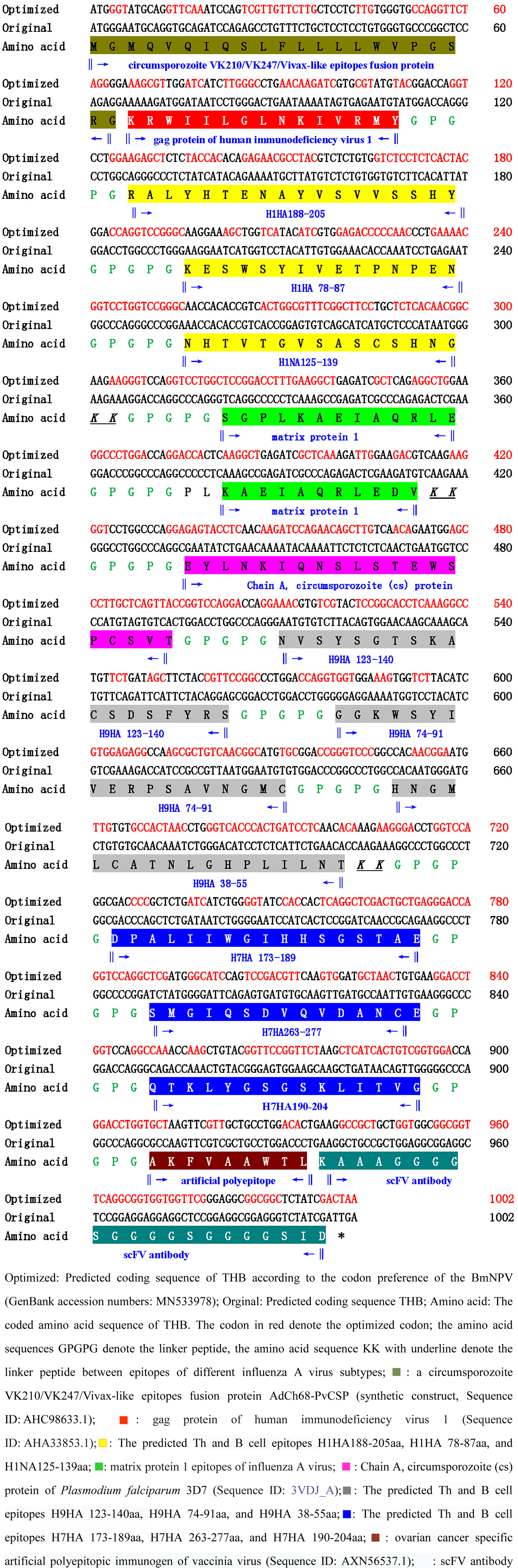

Supplement: Supplementary file 2 — Additional file 2: Figure S2. The synthesized THB sequence. [file 12985_2020_1388_MOESM2_ESM.tif]

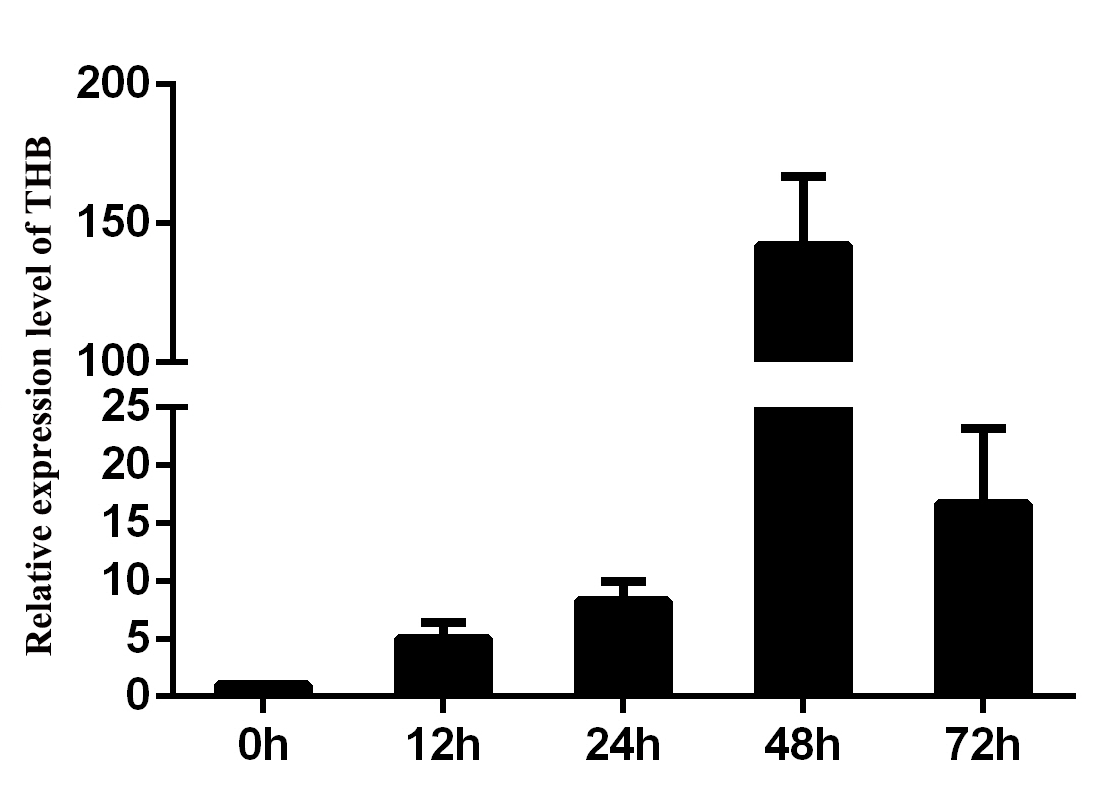

Supplement: Supplementary file 3 — Additional file 3: Figure S3. Level of THB expression at different stages in BmNPV-CMV/THB-P10/CTLT-infected HEK293T cells. The level of THB expression at 0, 12, 24, 48, and 72 h post-infection was determined by qRT-PCR. [file 12985_2020_1388_MOESM3_ESM.tif]

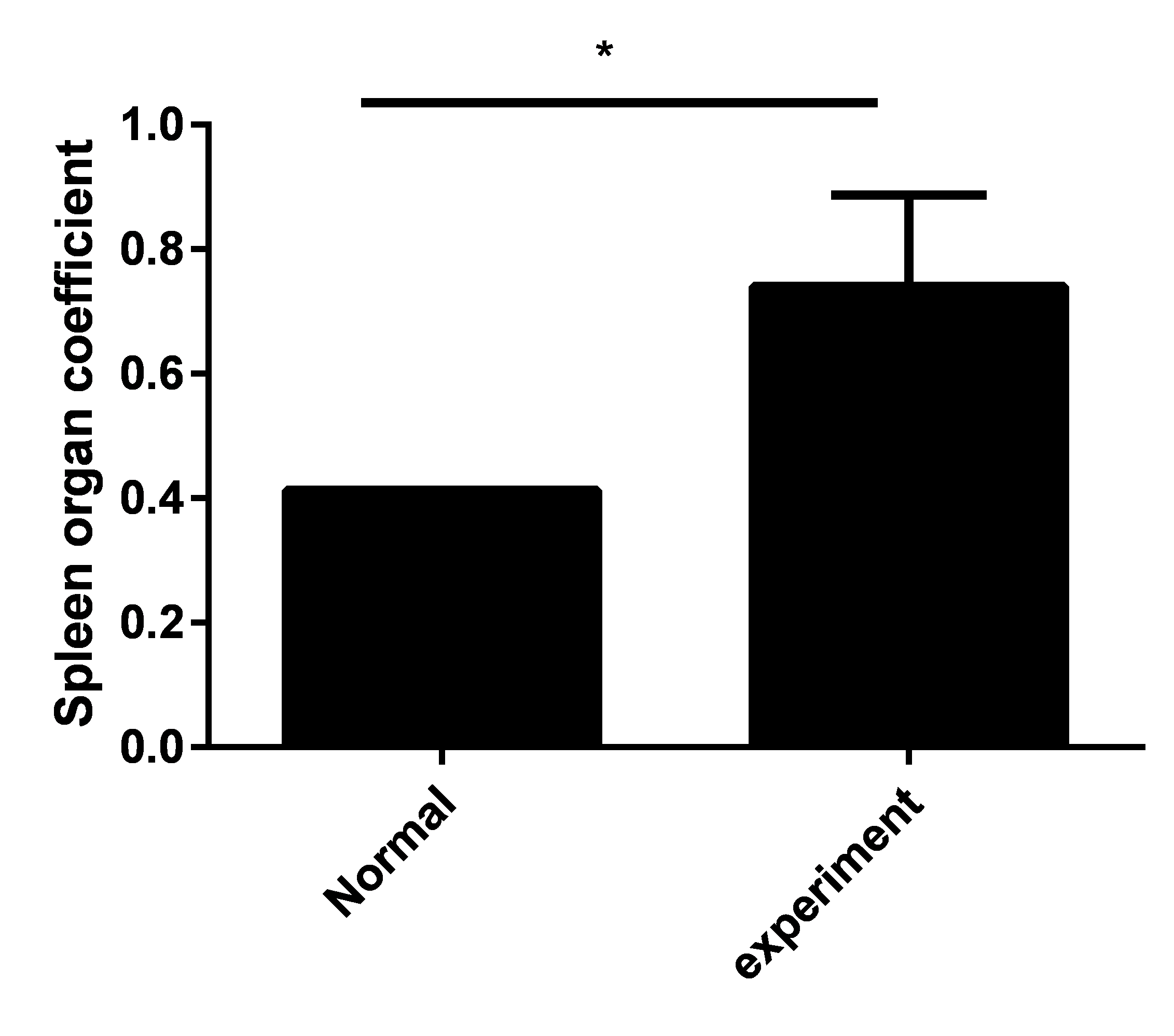

Supplement: Supplementary file 4 — Additional file 4: Figure S4. Effect of vaccination with BmNPV-CMV-THB-CTLT on the organ coefficient of the spleen. SPF BALB/c mice (approximately 20 g) were intraperitoneally injected with BmNPV-CMV/THB-P10/CTLT (100 μL) at a dose of 1012 TCID50, The organ coefficient of the spleens was investigated at 48 h post-injection. The unimmunized mice were used as a control. *p < 0.05. [file 12985_2020_1388_MOESM4_ESM.tif]
