# Supplementary material for: AIV polyantigen epitope expressed by recombinant baculovirus induces a systemic immune response in chicken and mouse models
Source: Virol J. 2020 Aug 5;17:121. doi: 10.1186/s12985-020-01388-w (PMC7403573; doi:10.1186/s12985-020-01388-w)
Supplement: Supplementary file 5 — Additional file 5: Table S1. The amino acid sequences and conservation of selected CTL epitopes. [file 12985_2020_1388_MOESM5_ESM.doc]

| The predicted T lymphocyte  epitopes | Position of epitope | Amino acid sequence | Conserved in AIV subtypes |
| --- | --- | --- | --- |
| H1HA 42-50 | 42-50 | CLLKGIAPLN | H1N1, H1N2 |
| H1HA 100-108 | 100-108 | ELREQLSSV | H1N1, H1N3 |
| H1HA 132-140 | 132-140 | VTAACSHAG | H1N1 |
| H9HA 19-27 | 19-27 | TLTENNVPV | H1N1 |
| H9HA 79-87 | 79-87 | YIVERPSAV | H1N1 |
| H9HA 124-132 | 124-132 | NVSYSGTSK | H1N1 |
| H7HA 26-34 | 26-34 | TLTERGVEV | H7N1, H7N2, H7N3, H7N4, H7N5, H7N7, H7N8, H7N9, H15N6, H15N9 |
| H7HA 192-200 | 192-200 | KLYGSGSKL | H7N1, H7N3, H7N4, H7N5, H7N7, H7N8, H7N9 |
| H7HA 40-48 | 40-48 | TVERTNIPR | H7N7, H7N9, H7N3, H7N4 |

**Table S1 The amino acid sequences and conservation of selected CTL epitopes**
