# Supplementary material for: AIV polyantigen epitope expressed by recombinant baculovirus induces a systemic immune response in chicken and mouse models
Source: Virol J. 2020 Aug 5;17:121. doi: 10.1186/s12985-020-01388-w (PMC7403573; doi:10.1186/s12985-020-01388-w)
Supplement: Supplementary file 6 — Additional file 6: Table S2. The amino acid sequences and conservation of selected THB epitopes. [file 12985_2020_1388_MOESM6_ESM.doc]

**Table S2 The amino acid sequences and conservation of selected THB epitopes**

| The predicted Th as well B cell epitopes | Position of epitopes | Amino acid sequence | Conserved in AIV subtypes |
| --- | --- | --- | --- |
| H1HA188-205 | 188-205 | RALYHTENAYVSVVSSHY | H1N1, H1N2 |
| H1HA 78-87 | 78-87 | KESWSYIVETPNPEN | H1N1, H1N2 |
| H1NA125-139 | 125-139 | NHTVTGVSASCSHNG | H1N1, H1N2 |
| H9HA 123-140 | 123-140 | NVSYSGTSKACSDSFYRS | H9N2 |
| H9HA 74-91 | 74-91 | GGKWSYIVERPSAVNGMC | H9N2 |
| H9HA 38-55 | 38-55 | HNGMLCATNLGHPLILNT | H9N2 |
| H7HA 173-189 | 173-189 | DPALIIWGIHHSGSTAE | H7N1, H7N2, H7N3, H7N6, H7N7, H7N8, H7N9 |
| H7HA263-277 | 263-277 | SMGIQSDVQVDANCE | H7N1, H7N2, H7N3, H7N7, H7N8, H7N9 |
| H7HA190-204 | 190-204 | QTKLYGSGSKLITVG | H7N1, H7N3, H7N4, H7N5, H7N6, H7N7, H7N8, H7N9 |
